# Nutritional status, alcohol-tobacco consumption behaviour and cognitive decline among older adults in India

**DOI:** 10.1038/s41598-022-25563-x

**Published:** 2022-12-06

**Authors:** Junaid Khan

**Affiliations:** 1 International Institute for Population Sciences, Deonar, Mumbai, 400088 India; 2 School of Public Health, Asian Institute of Public Health University, Bhubaneswar, 752101 India

**Keywords:** Risk factors, Public health, Epidemiology, Population screening

## Abstract

Cognition capacity is essentially age-dependent and it is associated with the overall well-being of an individual. The public health aspects of cognitive research primarily focus on the possible delaying of cognitive decline among the older adult population. In this context, using the most recent round of the Longitudinal Ageing Study in India, 2017–2018 data, this study examines the cognition capacity among older adults aged 45 and above subject to their nutritional health and health behaviour (tobacco and alcohol consumption). It is observed that almost one in every tenth individual (10%) above 45 years of age in India shows low cognition scores. Low cognition is much more prevalent among 60 + females than males. Around one-fifth of the underweight older adults (18%) demonstrate low cognition capacity among them. Of those older adults who consume only tobacco, 11% of them demonstrate low cognition than the rest. The partial proportional odds model estimation shows that older adults are at higher risk of developing low cognition with increasing age and beyond age 65, the individuals carry a critically higher risk to experience low cognition. The estimation also shows that with increasing age older adults are higher likely to experience poor cognition independent of nutritional status, but underweight older adults are comparatively more likely to experience low cognition followed by normal and overweight older adults. In terms of alcohol-tobacco consumption behaviour, older adults who consume both are more likely to experience low cognition with increasing age followed by ‘only alcohol consumers’, and ‘only tobacco consumers’.

## Introduction

Cognition refers to the combination of processes in an individual’s brain. It determines the capacity to learn, remember, make judgments, reasoning, intelligence and other mental functions whereas, an impaired or poor cognition critically impacts one’s overall well-being and health^1,2^. Cognition capacity is essentially age-dependent, declines during old age and ranges from mild cognitive impairment to dementia, a severe form of cognitive impairment^3,4^. And the overall physical health of older adults which is defined by mobility, disability, frailty, cardiovascular events and other bodily events is related to the cognitive status of the person^5–11^.

Ageing is inevitable and healthy ageing is one of the most important public health issues globally and India is not an exception to the issues of population ageing with a growing number of older persons in the total population; 8% of the total population consists of older adults in India^12^. As per the 2011 census estimates, India is home to 93 million older adults (60 +) and it is projected to be the second highest contributor to the older population globally after China with a older (60 +) population of 323 million by the end of 2050^3^.

As cognitive capacity is associated with the overall well-being of a person; thus, plays an important role in healthy ageing^13^. In this direction, the cognition research in the public health domain keenly focuses on possible delaying the decline in cognition among older persons^14^. The active model of ‘cognition reserve’ hypothesises the role of experience and argues that education level, career achievements and social activities determine the level of cognition among individuals^15^. These factors can play a protective role in delaying cognitive aging^15–17^. The cognitive function of an individual is exclusively determined by genetic and socio-demographic factors also^18^. Previous studies suggest that socio-economic factors, health status and health behaviour are associated with the cognitive performance of an individual^19^. As socio-demographic factors; age, sex, educational qualification, economic condition, place of residence is found to be associated with better cognitive functioning^20–23^.

On the other hand, health behaviours like smoking and drinking also play a role in significantly affecting the cognitive capacity among older adults^24,25^. It is found that the risk of dementia is higher among heavy drinkers^26^. Due to the direct and indirect effects of alcohol on the brain, excessive drinking over a prolonged period increases the risk of brain damage which may lead to Wernicke-Korsakoff syndrome and alcoholic dementia^27,28^. The alcohol-associated brain damage and dementia are partially reversible when consumption of alcohol is reduced or stopped^29,30^. Body Mass Index (BMI) is another important factor that affects cognitive function among older adults^31^. Previous studies report that elevated BMI is associated with lower structural integrity between the frontal and temporal lobes in a brain which is likely to affect the cognitive capacity among older people^32,33^.

Although the selling of alcohol is totally banned in some of the states in India^34^; yet, estimates through different national-level surveys provide evidence of alcohol consumption across all the states of India^35–37^. In India, across different social classes and among the tribal population, the consumption of alcohol prevails in their culture and spirituous liquors like arrack, toddy and rice beers are commonly used^38–40^. Alcohol consumption is also associated with different social celebrations, such as births, marriages and festivals across different population groups in India^38,41^. Introduced by the Portuguese in the seventeenth century, tobacco consumption became socially accepted and embedded within the traditions of cultural rituals^42^. Tobacco usage had shown a different dynamics in India and evolved in two different forms- smoked and chewing tobacco whereby ‘khaini' is identified as the locally prepared chewable tobacco and ‘bidi’ and ‘chutta’ as the smoked tobacco^42^.

In this context, the aim of this study is to examine the cognitive capacity among Indian older adults aged 45 and above subject to their BMI status and their tobacco and alcohol consumption behaviour using the most recent round of the LASI, wave 1 survey data. The socio-economic and demographic characteristics of the respondents are controlled to examine the current status of the cognitive capacity in the study population. The Composite International Diagnostic Interview (CIDI) scale is used to measure the cognitive capacity of individuals. The CIDI scale provides the standard measure of the cognitive capacity of an individual^35^.

## Data

This study utilises the Longitudinal Ageing Study in India (LASI) wave 1 survey data. The data is publicly available through https://www.iipsindia.ac.in/content/lasi-wave-i subject to a data request. Thus, this study requires no ethical approval further. LASI wave 1 is essentially the most recent survey data on older population and provides the necessary information on ageing demography and most importantly the geriatric health information of Indian older adults. LASI is India’s first ever longitudinal ageing survey. LASI wave 1 was conducted from April, 2017 to December, 2018 across 35 Indian states and union territories. LASI is a nationally representative survey and surveyed 72,250 older adults aged 45 and above across India from 42,949 LASI age-eligible (households with at least one member aged 45 and above) households. Of the total individuals, there are 31,464 individuals aged 60 and above. LASI adopted a multistage stratified area probability cluster sampling design. The details of the sampling design and survey instruments are mentioned elsewhere^35^.

### Analytical sample

This study exclusively includes those individuals of age 45 and above and not their spouses irrespective of their age. Of the total 72,250 sampled individuals, a total of 6,688 individuals of age less than 45 years are dropped. Of the total 65,562 individuals aged 45 and above, the domain-wise complete information on cognition is available for 57,431 individuals and the composite score of cognition is computed for these many individuals. The height and weight information are available for the 59,073 individuals. The combined alcohol and tobacco information is available for 65,008 individuals. Given the completeness of the information on cognition, the anthropometric information on body mass index (BMI) is not available for 4766 individuals. So, the analytical sample size of this study is 52,665. LASI is essentially a large-scale survey data but there are instances where there are missing observations in the dataset across variables. Considering this fact, a complete case analysis approach^43^ has been adopted to overcome the analytical challenges whereby all the estimates produced in this study are survey weight adjusted. Additionally, as the dataset is large enough in sample size, the estimation does not pose any threat of unbiased estimation on this account due to the sample size. Sample size by the categories of the variables is shown in Online Appendix S1.

### Outcome variable

The main outcome variable of this study is cognitive capacity among individuals aged 45 and above. The cognitive capacity of the individuals is measured in terms of composite index which is the combined score of memory (total word recall), orientation, arithmetic function, executive function and naming of objects. The raw scores lie between the values 0 and 41. A score in the range of 0–15 is defined as the low score, a score in between 16–30 as medium and a score in the range of 31–41 as high score.

- Memory score is the total word recall by the surveyed individuals which is the sum of the immediate and delayed word recall scores^35^.
- Orientation particularly refers to the orientation of time and place^35^.
- Verbal fluency of an individual is the cognitive function and informs the retrieving capacity of memory^35^.
- Individual’s arithmetic function skills are the numeric ability of backward counting, serial 7 and computation skills^35^.
- Executive function refers to the individual’s mental processes that enable the person to successfully plan, focus attention, remember instructions, and juggle multiple tasks^35^.
- Naming of objects assessed the individual’s vocabulary knowledge along with the crystallized intelligence which necessarily refers to the ability to use skills, knowledge and experience^35^.

### Key exposure variables

#### BMI status

The body mass index of an individual is measured in terms of their height and weight and defined as weight (in Kilograms) divided by height (in meters) square. A person is identified as underweight if his/her BMI score ≤ 18.5 kg/m^2^. Similarly, a BMI score ≥ 25 kg/m^2^ is defined as overweight and obesity as BMI score ≥ 30 kg/m^2^^44^.

#### Alcohol-tobacco consumption behaviour

Tobacco and alcohol consumption behaviour of the individuals are combined together and a composite variable is created. The variable is defined as- ‘consumes nothing’, ‘consumes only tobacco (smoke or smokeless)’, ‘consumes only alcohol’ and ‘consumes both’.

#### Covariates

The set of covariates includes socio-economic, demographic characteristics of the individuals. These include –type of residence (Rural/Urban), sex (Male/Female), age (45–59 years/60 and above), living arrangement (Living alone/ Living with Spouse and other/ Living with Spouse and Children/ Living with Children and other/ Living with other exclusively), marital status (Currently Married/ Widowed/ Other), social caste (Scheduled Caste/ Scheduled Tribe/Other Backward Class/None), educational attainment (Up-to Primary/ Secondary/ Higher and above), MPCE quintiles (Poorest/Poor/Middle/Richer/Richest) and regions (North/ East/ North-East/ South/ Central/ West).

## Methodology

The primary analysis includes simple frequency statistics followed by bivariate analysis. Using bivariate cross-tabulation, the prevalence of low, medium (not shown) and high cognition is computed by background characteristics of the individual subject to their BMI status and alcohol-tobacco consumption behaviour pattern. An empirical investigation of the data involves a series of regression analyses suitable for the ordinal type of response variable which is discussed below.

The response variable is a categorical variable and identifies the cognition capacity among older adults and is coded as 0 (low), 1 (medium) and 2 (high). There are several factor variables used as the covariates and BMI status of the individuals and the tobacco and alcohol consumption behaviour among the older adults are assumed to be the two key exposure variables while predicting the cognition capacity of the individuals.

Given the choice of the outcome variable and its categories (low, medium and high), generalised ordered logistic regression analysis is applied to investigate how BMI status of the individual and their tobacco and alcohol consumption behaviour influence the cognition among older adults aged 45 and above. As the parallel regression assumption is checked; both the likelihood-ratio test of proportionality of odds across the response variable's categories and the ‘Brant test’ provides the evidence that the parallel regression assumption does not hold true, thus a generalised ordered logistic model is applied.

The generalised ordered logit model can be specified as

$$\mathrm{Pr}\left({Y}_{i}>j\right)=g\left(X{\beta }_{j}\right)=\frac{\mathrm{exp}({a}_{j}+{X}_{i}{\beta }_{j})}{1+\left[\mathrm{exp}({\alpha }_{j}+{X}_{i}{B}_{j})\right]}, j=\mathrm{1,2},\dots ,M-1$$

where M is the number of categories of the ordinal outcome variable. From the above equation, the probability of Y taking a value ‘j’ can be easily deduced by the following-

$$\mathrm{Pr}\left({Y}_{i}=j\right)= g\left({X}_{i}{\beta }_{j-1}\right)-g\left({X}_{i}{\beta }_{j}\right) j=2,\dots , M-1$$

As the parallel line assumption is violated, the estimates for each of the variables from the unconstrained model are further checked using a Wald test to see whether the coefficients of the variable differ significantly across the equations. A statistically insignificant Wald test confirms the equal effects of the particular variable across the equations. Additionally, the global Wald test of the final model with constraints against the unconstrained model provides the statistical evidence supporting the final constrained model. As the partial proportional model gives statistically insignificant chi-square value [chi^2^ (14) = 11.77; p-value = 0.6249], the estimates from the partial proportional odds model are considered to be final. Online Appendices S1 and S2 show the results from the ordered logistic and generalised ordered logistic model estimation respectively.

## Results

The demographic profile of the study population is shown in Online Appendix S1. Of the total sample (aged 45 and above), 53.7 percent are females. Almost half (47%) of the individuals are above 60 years of age. The majority (65%) of the population is living in rural areas and 76 percent are currently married. Only, 18% of the older adults have completed higher education. Around 4% of the geriatric population is living alone and 39% percent belong to the two socially excluded class-scheduled caste and scheduled tribe categories. Around two-fifths of the study population belongs to the lowest two MPCE quintiles.

### Prevalence of underweight, overweight and obesity among older adults

Almost 50% of the older population in India are found malnourished during 2017–2018 (Online Appendix S2). Approximately, 21% of the population above 45 years of age is underweight and 7% of the population is obese. The prevalence of being underweight is comparatively higher among men and women aged 60 years and above. On the other hand, prevalence of overweight and obesity is higher among women than men, and the prevalence is substantially higher among female older adults aged 45–59 years (Online Appendix S2).

### Consumption of tobacco and alcohol among older adults

Approximately 12% of the population above 45 years of age consume both tobacco and alcohol, 26% consume only tobacco and 3% consume only alcohol (Online Appendix S2). Twenty one percent of the population use smokeless tobacco compared to 14% older adults who are involved in consuming smoked tobacco. Three out of every 10 individuals consuming alcohol are heavy drinkers (Online Appendix S2). Prevalence of tobacco and alcohol consumption is found to be substantially higher among men and the prevalence is comparatively higher among individuals between 45 and 59 years of age than those above 60 years of age. An exception is observed in terms of smokeless and smoked tobacco and the prevalence of consumption of tobacco (smoked, smokeless) is observed higher among females above 60 years of age than those between 45 and 59 years of age.

### Cognition status among older adults

It is observed that almost one in every tenth individual (10%) above 45 years of age has a low cognition score (Online Appendix S2). Among males; compared to 29% of the older adults (45–59 years), only 16% of the individuals age 60 and above show high cognition scores. And among 60 + females, only 6% show high cognition than 14% of the older adult females (45–59 years). Low cognition is much more prevalent among 60 + females (22%) than males (8%) (Online Appendix S2).

### Cognition among older adults by BMI status and background characteristics

Table 1 shows the cognition status among the geriatric population with respect to their nutritional status and background characteristics. A distinct pattern of cognition has been observed among older adults of different population characteristics, wherein individuals who are underweight have low cognition scores in general whereas those who are overweight and obese have a comparatively high cognition in terms of the measured cognition score. The older population, residing in urban areas have a higher proportion of individuals who demonstrate high cognition scores irrespective of their nutritional status, and in general, the cognition score is higher among males compared to females across different BMI categories.

**Table 1:** Prevalence of low and high cognition among older adults aged 45 and above by BMI (nutritional) status and background characteristics of the individuals, LASI, Wave1, 2017–2018.

Background characteristics	BMI status					
	Underweight		Overweight		Obese	
	Low	High	Low	High	Low	High
**Residence**						
Rural	19.91	5.91	6.14	18.02	5.92	16.52
Urban	9.18	10.28	3.09	30.11	3.98	30.09
**Sex**						
Male	10.64	10.45	1.28	34.64	0.68	35.98
Female	25.64	2.88	7.06	16.19	6.06	21.04
**Age**						
45–59	11.39	8.84	3.31	29.19	2.28	28.75
60 +	22.75	5.1	6.73	15.78	8.93	17.77
**Living arrangement**						
Living alone	25.43	4.58	9.83	19.93	11.45	16.25
Living with spouse and other	17.12	6.63	4.3	21.36	1.58	23.97
Living with spouse and children	12.8	8.77	2.7	28.1	3.36	28.27
Living with children and other	28.56	2.8	10.69	11.91	10.96	14.92
Living with other	23.59	3.75	8.87	17.04	8.04	19.77
**Marital status**						
Currently married	13.83	8.26	3.02	26.96	3.06	27.16
Widowed	30.24	2.62	11.11	10.46	11.42	13.49
Other	8.19	6.46	6.27	31.23	5.03	34.03
**Caste**						
Scheduled caste	21.74	4.87	6.13	17.2	6.43	12.62
Scheduled tribe	28.93	3.74	6.74	18.21	4.68	12.23
Other backward class	14.71	7.94	4.57	24.68	6.03	28.6
None	14.1	7.93	3.81	27.06	2.71	25.25
**Education**						
Upto primary	6.54	10.12	1.5	17.93	0.93	21.78
Secondary	0.96	25.83	0.13	36.01	0.32	33.47
Higher and above	0.01	22.2	0.13	55.00	1.00	59.17
**MPCE**						
Poorest	22.43	6.08	5.39	16.51	7.13	14.77
Poor	19.46	5.26	3.8	19.39	3.56	17.14
Middle	15.89	7.26	6.21	18.53	4.01	20.37
Rich	14.31	7.93	5.16	23.23	7.42	31.35
Richest	16.57	7.13	3.19	36.32	2.51	31.38
**Region**						
North	18.7	5.47	5.91	17.31	4.84	19.84
East	21.18	5.44	2.88	24.19	2.98	18.44
North-east	21.98	9.57	5.29	31.03	4.03	28.19
South	12.13	10.88	3.28	30.97	3.21	37.99
Central	14.81	5.21	4.57	24.54	7.09	27.92
West	20.8	4.91	6.52	21.37	5.16	18.96
Total	18.4	6.53	4.7	23.73	4.76	24.66

Of those who are underweight and living alone, one-fourth of them show low cognition scores and only 5% of them demonstrate high cognition scores. It is observed that the underweight individuals and those who are living without spouse show a comparatively higher prevalence of low cognition. This situation is in contrast and comparatively better among the overweight and obese older adults. The older adults in India also show a variation in their cognition status by their BMI and marital status and it is found that those who are widowed and underweight show a quite high prevalence (30%) of low cognition followed by 11% (almost equal) among overweight and obese widowed individuals. By social class, scheduled caste (22%) and scheduled tribe (29%) older persons who are underweight show a comparatively higher prevalence of low cognition. On the contrary, except the two socially excluded caste groups, rest of the older adults who are either overweight or obese demonstrate a higher prevalence of high cognition. Educational attainment of the older adults shows a clear variation and it is observed that those who are higher educated carry better cognition independent of their nutritional status. For example, the underweight older population who are more than secondary educated, 22% of them show high cognition compared to 10% among the ‘up to primary’ educated. The prevalence of high cognition is as high as 55% among the overweight and 59% among the obese when the individual is higher and above educated. Economic status of the older persons shows a clear gradient (declining trend) of low cognition among the underweight and more than one-fifth (22%) of the underweight older adults from the poorest MPCE quintile are observed to be of low cognition status compared to 17% in the richest MPCE quintile. On the other hand, overweight and obese older adults across the higher MPCE quintiles show higher prevalence of high cognition. Subject to BMI status, the regional prevalence of low cognition among the underweight older adults shows that except the South and Central region of India, all the other regions carry high prevalence of low cognitive scores among the older adults. Approximately, 22% of the underweight older population from North-East India show low cognitive scores followed by 21% in East India and in Western India. The prevalence of high cognition is observed highest (38%) among the obese older adults in the South India among all the regions.

### Cognition among older adults by alcohol and tobacco consumption and by background characteristics

Table 2 presents the prevalence of low and high cognition subject to substance use like alcohol and tobacco consumption across the sub-population. Similar to the findings of cognition status by nutritional status in the study population, it is also found that the prevalence of high cognition score is higher among older adults living in urban areas compared to their counterparts in rural areas for each of the categories of alcohol-tobacco consumption types. The gender pattern shows that males who consume both demonstrate a prevalence of 8% of low cognition; whereas, the prevalence is as high as 32% among females who consume both. The age pattern of prevalence reflects that the 60 + population with habits of alcohol and or tobacco consumption carry a significantly higher burden of low cognition than their counterparts in the age group of 45–59.

**Table 2:** Prevalence of low and high cognition among older adults aged 45 and above by alcohol and tobacco consumption behaviour and background characteristics of the individuals, LASI, Wave1, 2017–2018.

Background characteristics	None		Only tobacco		Only alcohol		Both	
	Low	High	Low	High	Low	High	Low	High
**Residence**								
Rural	12.85	10.95	12.74	10.78	13.6	15.73	10.56	13.12
Urban	4.62	26.53	6.62	20.47	1.31	33.08	4.18	26.79
**Sex**								
Male	3.41	29.67	5.06	18.06	5.15	25.12	7.48	17.04
Female	12.24	11.78	21.67	4.39	29.23	3.8	32.42	2.51
**Age**								
45–59	5.21	21.79	6.54	17.28	5.22	27.81	6.35	20.94
60 +	15.11	10.79	15.53	9.09	15.92	12.13	12.17	10.92
**Living arrangement**								
Living alone	17.68	10.39	23.31	7.31	28.25	19.52	12.9	8.97
Living with Spouse and other	8.88	15.59	10.44	10.18	10.6	18.21	8.19	12.3
Living with Spouse and Children	5.87	20.92	7.21	16.06	5.52	24.95	7.71	19.14
Living with Children and other	18.01	8.58	21.63	6.48	26.11	3.28	18.3	5.65
Living with other	17.18	10.92	21.82	7.71	22.93	22.34	12.16	12.91
**Marital status**								
Currently Married	6.59	19.74	7.88	14.87	6.48	23.62	7.81	17.8
Widowed	19.35	7.48	23.41	5.59	27.56	4.16	19.74	5.77
Other	7.82	19.68	13.31	14.23	22.15	27.1	4.28	8.38
**Caste**								
Scheduled Caste	13.2	10.3	14.81	8.51	6.46	21.39	10.58	11.43
Scheduled Tribe	19.79	8.4	17.81	8.09	27.5	8.37	22.35	9.13
Other Backward Class	8.71	17.71	10.35	13.66	5.1	22.35	5.06	17.89
None	6.9	21.28	7.89	16.83	3.2	33.72	3.83	25.42
**Education**								
Upto Primary	3.4	14.62	3.76	15.09	2.17	18.77	3.8	13.25
Secondary	0.63	33.39	0.45	29.93	0.12	37.59	0.47	33.24
Higher and Above	0.31	54.50	NA	47.78	NA	50.38	0.13	48.44
**MPCE**								
Poorest	12.52	10.27	14.85	10.73	19.46	12.31	13.43	10.98
Poor	11.14	13.26	11.97	9.71	13.45	18.57	12.23	11.37
Middle	10.5	14.34	9.33	13.74	9.04	18.42	8.42	16.32
Rich	7.32	19.57	11.9	13.65	5.94	19.58	5.22	21.37
Richest	7.59	26.29	7.97	18.58	1.58	36.28	4.83	22.94
**Region**								
North	11.95	12.02	11.91	10.59	2.99	27.04	6.42	15.39
East	11.71	13.61	10.84	11.99	18.62	19.59	12.87	15.93
North-east	11.95	22.84	14.53	18.45	8.12	17.13	9.32	22.41
South	5.5	23.16	8.64	20.18	10.41	21.35	7.09	13.56
Central	7.94	19.51	15.19	11.07	8.68	13.78	9.33	17.54
West	11.28	15.48	11.53	11.18	9.95	21.34	8.57	16.87
Total	9.84	16.65	11.48	12.77	9.97	20.85	9.2	16.04

By living arrangement, the prevalence of low and high cognition among older adults show a variation and those who are either living alone or living without a spouse carry a higher prevalence of low cognition than their counterparts. The prevalence of low cognition is substantially higher among those ‘living alone’ older adults who either consume tobacco (23%) or alcohol (28%). Older adults who are widowed show comparatively higher prevalence of low cognition independent of alcohol, tobacco consumption behavior and almost one-fourth of the widowed older adults carry low cognition when exposed to alcohol or tobacco consumption. Similar to the pattern of cognition by nutritional status, the scheduled tribe (ST) population carries the highest proportion of older adults with low cognition score irrespective of their alcohol and tobacco consumption status. Educational attainment of the study population also shows a distinct pattern of low and high cognition prevalence and older adults who are higher educated demonstrate better cognition scores than their counterparts with lower educational qualification. And the prevalence of high cognition is highest (55%) among those individuals who are higher educated and do not consume tobacco or alcohol. Older adults from the higher MPCE class show better cognition status among them and it is the richest class show highest prevalence of high cognition independent of their alcohol or tobacco consumption behavior. On the other hand, older adults from the poorest MPCE class and exposed to some forms of substance use demonstrate a comparatively higher prevalence of low cognition. Approximately, 20% of older adults from the poorest MPCE quintile who consume only alcohol have low cognition score followed by 15% among those older adults who use only tobacco and a prevalence of 13% among those who consume both alcohol and tobacco. On the other hand, almost 8% of the older population show low cognition score who belong to the richest MPCE quintile and consume only tobacco. It is also found that 13% of older adults from East India who consume both tobacco and alcohol have low cognition score followed by North-East and Central India. Moreover, 19% of the older adults form the East India, who consume only alcohol are found to have a low cognition score and 15% of older adults from North-East India consuming only tobacco are found to have low cognition score.

### Regression results

Table 3 presents the results from the partial proportional odds model estimation. Result provides the coefficient values for the two key exposure variables along with the other covariates- the socio-economic and demographic characteristics of the individuals. Given the three different categories of cognition (low, medium and high), there are two different equations being estimated. In the first equation, medium and high cognition are compared against low cognition whereas high cognition is compared against low and medium in the second equation. Table 3 provides the details of the two sets of coefficients, statistical significance (p-value) and the 95% confidence intervals.

**Table ﻿3:** Partial proportional odds model estimation of cognition score of older adults aged 45 and above, India, LASI Wave 1, 2017–2018.

Background characteristics	Low vs medium and high			Low and medium vs high		
	AOR	p-value	95% CI	AOR	p-value	95% CI
**BMI status**						
Normal						
Underweight	− 0.38	< 0.01	− 0.44 : 0.32	− 0.38	< 0.01	− 0.44 : 0.32
Overweight	0.37	< 0.01	0.27 : 0.47	0.20	< 0.01	0.14 : 0.27
Obese	0.29	< 0.01	0.21 : 0.37	0.29	< 0.01	0.21 : 0.37
**Substance use**						
No tobacco, no alcohol						
Tobacco only	− 0.08	0.013	− 0.14: 0.03	− 0.08	< 0.01	− 0.14 : 0.03
Alcohol only	− 0.13	0.011	− 0.23 : 0.03	− 0.13	0.01	− 0.23 : 0.03
Both	− 0.18	< 0.01	− 0.25 : 0.11	− 0.18	< 0.01	− 0.25 : 0.11
**Age**						
45–59						
60 +	− 0.85	< 0.01	− 0.92 : 0.78	− 0.51	< 0.01	− 0.56 : 0.45
**Residence**						
Rural						
Urban	0.44	< 0.01	0.39 : 0.49	0.44	< 0.01	0.39 : 0.49
**Sex**						
Male						
Female	− 0.67	< 0.01	− 0.75 : 0.59	− 0.44	< 0.01	− 0.50 : 0.38
**Living arrangement**						
Living alone						
Living with spouse and other	− 0.13	0.218	− 0.34 : 0.08	− 0.13	0.22	− 0.34 : 0.08
Living with spouse and children	0.01	0.941	− 0.19 : 0.21	0.01	0.94	− 0.19 : 0.21
Living with children and other	0.07	0.241	− 0.05 : 0.19	0.07	0.24	− 0.05 : 0.19
Living with other	− 0.15	0.051	− 0.29 : 0.00	− 0.15	0.05	− 0.29 : 0.00
**Marital status**						
Currently married						
Widowed	− 0.59	< 0.01	− 0.78 : 0.40	− 0.32	< 0.01	− 0.52 : 0.13
Other	− 0.46	< 0.01	− 0.71 : 0.20	0.16	0.18	− 0.07 : 0.38
**Caste**						
Scheduled caste						
Scheduled tribe	− 0.58	< 0.01	− 0.67 : 0.48	− 0.43	< 0.01	− 0.53 : 0.33
Other backward class	0.16	< 0.01	0.10 : 0.22	0.16	< 0.01	0.10 : 0.22
None	0.13	0.009	0.03 : 0.23	0.01	0.80	− 0.07 : 0.09
**Education**						
No education						
Upto primary	1.32	< 0.01	1.25 : 1.39	1.32	< 0.01	1.25 : 1.39
Secondary	2.76	< 0.01	2.51 : 3.01	2.19	< 0.01	2.11 : 2.26
Higher and above	3.49	< 0.01	2.92 : 4.06	2.88	< 0.01	2.79 : 2.97
**MPCE**						
Poorest						
Poor	0.13	0.002	0.05 : 0.22	0.03	0.52	− 0.06 : 0.11
Middle	0.20	< 0.01	0.13 : 0.27	0.20	< 0.01	0.13 : 0.27
Rich	0.35	< 0.01	0.25 : 0.44	0.23	< 0.01	0.15 : 0.31
Richest	0.40	< 0.01	0.32 : 0.47	0.40	< 0.01	0.32 : 0.47
**Region**						
North						
East	0.01	0.865	− 0.09 : 0.10	0.19	< 0.01	0.10 : 0.27
North-east	0.00	0.973	− 0.12 : 0.12	0.44	< 0.01	0.34 : 0.54
South	0.23	< 0.01	0.13 : 0.33	0.53	< 0.01	0.45 : 0.61
Central	0.04	0.619	− 0.10 : 0.18	0.37	< 0.01	0.25 : 0.49
West	− 0.39	< 0.01	− 0.49 : 0.28	− 0.12	0.01	− 0.21 : 0.03

**Figure 1:**
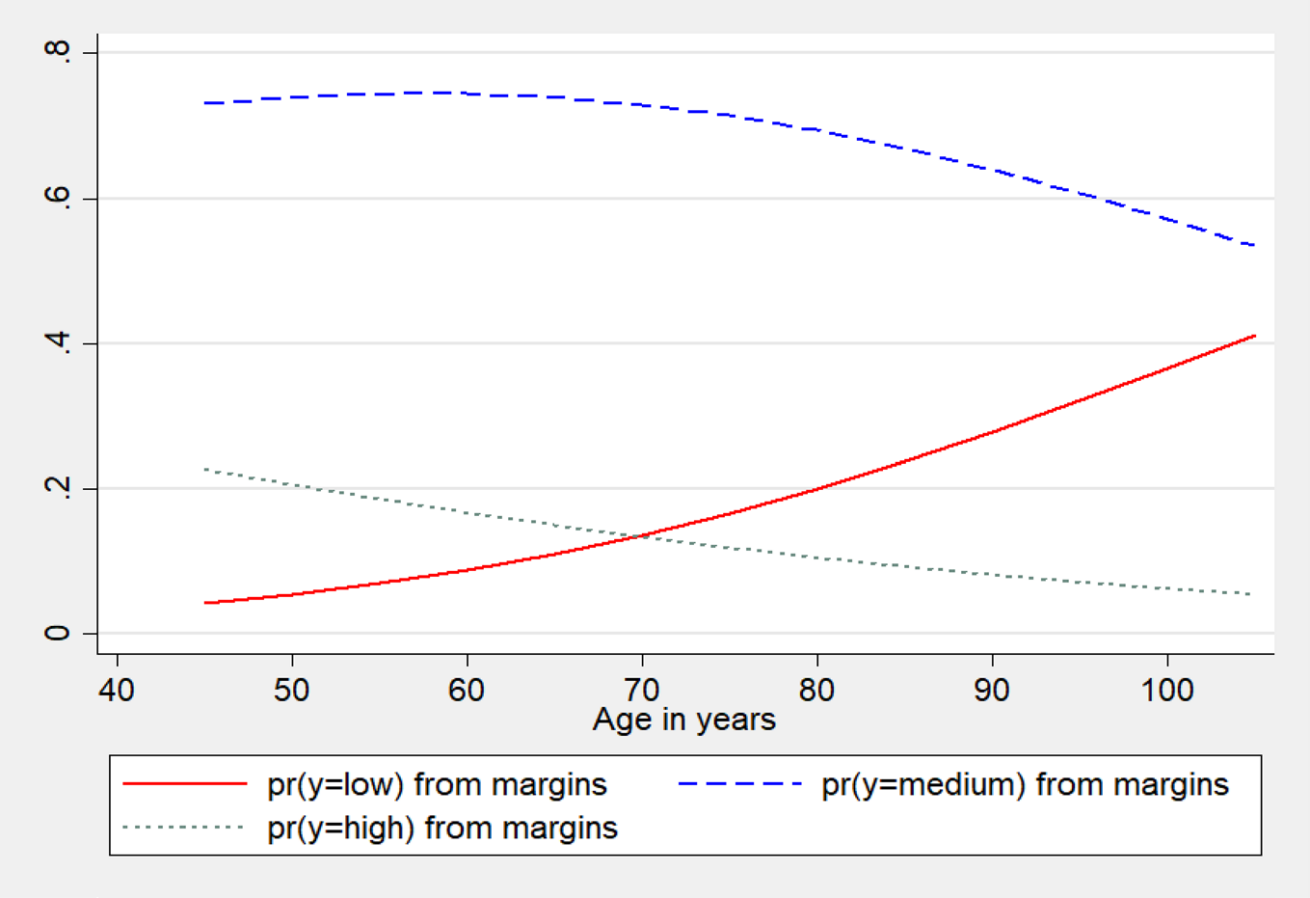
Average marginal effects of age on low, medium and high cognition among older adults, LASI, Wave 1, 2017–2018.

Cognition shows a clear age pattern among Indian older adults and it is found that individuals of higher age are more likely to experience low cognitive capacity among them. And compared to the individuals of age 45–59, older adults of age 60 or more are higher likely to experience lower cognition (Table 3). At the same time, the effects of age are apparently different across the equations and essentially the estimated beta coefficient is found larger from the first equation (low vs medium and high), which shows that the effect is higher when the 60 + population is compared against the 45–59 aged population to experience low cognition than medium or high cognition. The larger beta coefficient also shows that the probability to experience low cognition is higher among the 60 + population compared to the 45–59 year age group of older population. It is also additionally examined, the adjusted marginal effects (AMEs) across ages among the individuals (Fig. 1). The red line (solid) shows the predicted probabilities of low cognition, the blue line and green line show the predicted probabilities of medium and high cognition respectively in the study population with increasing age. The red line shows a monotonic increase in the probability to experience low cognition with increasing age and it is also apparent that the rate is higher above age 60 among older adults. This means that older persons are at higher risk of experiencing poor cognition in higher ages.

### BMI status and cognition

**Figure 2:**
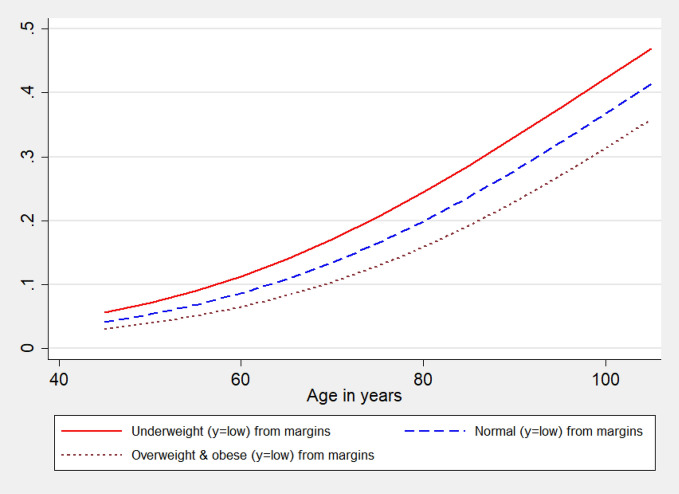
Average marginal effects of age on low cognition by body mass index (BMI) among older adults, LASI, Wave 1, 2017–2018.

BMI status of older persons show highly statistically significant association with their cognition capacity but the estimated coefficients for the underweight category do not show any significant change across the equations and the effects are exactly equal. Thus, compared to those older adults with normal BMI, underweight older adults are highly likely to be in the same or lower cognition category. This means that the underweight individuals are highly likely to have lower (low vs medium and high: β = − 0.38; p-value < 0.01; low and medium vs high: β = − 0.38; p-value < 0.01) or same cognition compared to those who are of normal BMI status. Similar to underweight, obese persons also show the same but positive effects across the equations. And compared to normal persons, obese are higher likely to be in the current or in the higher cognition category. Contrary to the underweight and obese, the overweight category shows varying effects across the equations and shows a stronger association (β = 0.37; p-value < 0.01) in the low vs medium and high cognition comparison equation. But in general, it is found that overweight individuals have a higher chance to have a higher cognition capacity compared to normal individuals. Figure 2 depicts the average marginal effects (AMEs) by BMI status on experiencing low cognition among older adults aged 45 and above. The ‘red line’ portrays the probability of experiencing low cognition by the underweight individuals and similarly the ‘blue line’ shows the corresponding probabilities for the normal individuals whereas the ‘maroon line’ represents the overweight and obese. Although cognition is highly age-dependent and individuals are most certainly exposed to low cognition with ageing, i.e., the probability to experience low cognition is inevitably higher in the higher ages but underweight individuals are exposed to higher chances to experience low cognition followed by normal and overweight and obese individuals (Fig. 2).

**Figure 3:**
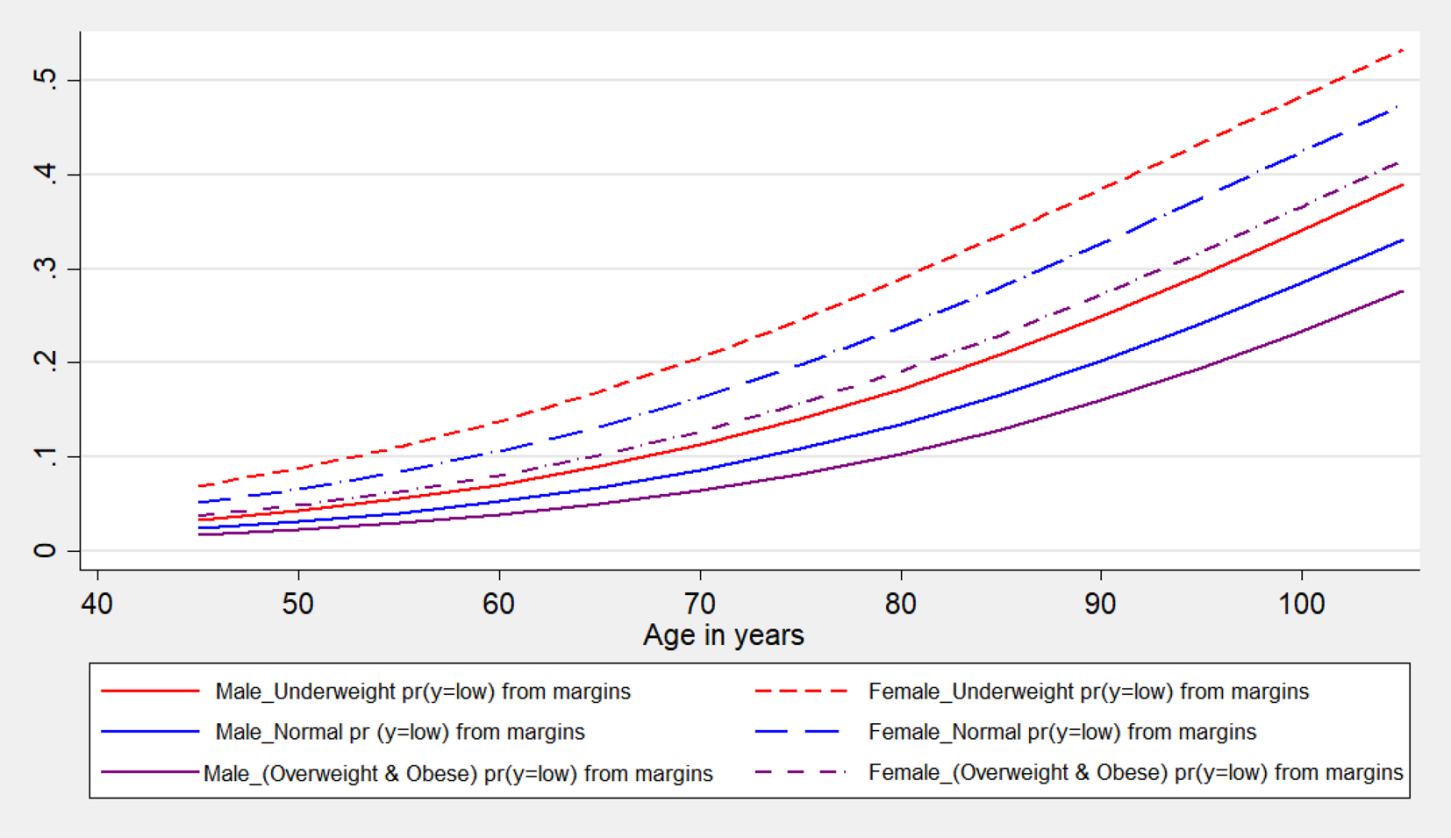
The gender pattern of average marginal effects of age on low cognition by body mass index (BMI) among older adults, LASI, Wave 1, 2017–2018.

The probability to experience low cognition among the individuals is further checked by sex and BMI status and is shown in Fig. 3. It is found that females of different ages show higher chances to experience low cognition than the males of the same age independent of the BMI status. But essentially, BMI status shows a variation in the corresponding probability among both males and females. And it is the underweight females who carry the highest risk to experience low cognition than all the other sub-groups. While overweight and obese males carry the lowest chance to experience low cognition (Fig. 3).

### Alcohol-tobacco consumption and cognition

Substance use among the individuals also shows a statistically significant association across the equations and the effects are found equal for all the categories of substance use patterns. From the estimated beta coefficients across the equations, it is pertinent that individuals who consume both tobacco and alcohol are the most likely (for both the equations, β = − 0.18; p-value < 0.01) to experience lower cognition than the reference group of individuals and the likelihood is found the lowest among those who consume only tobacco (for both the equations, β = − 0.08; p-value < 0.01).

**Figure 4:**
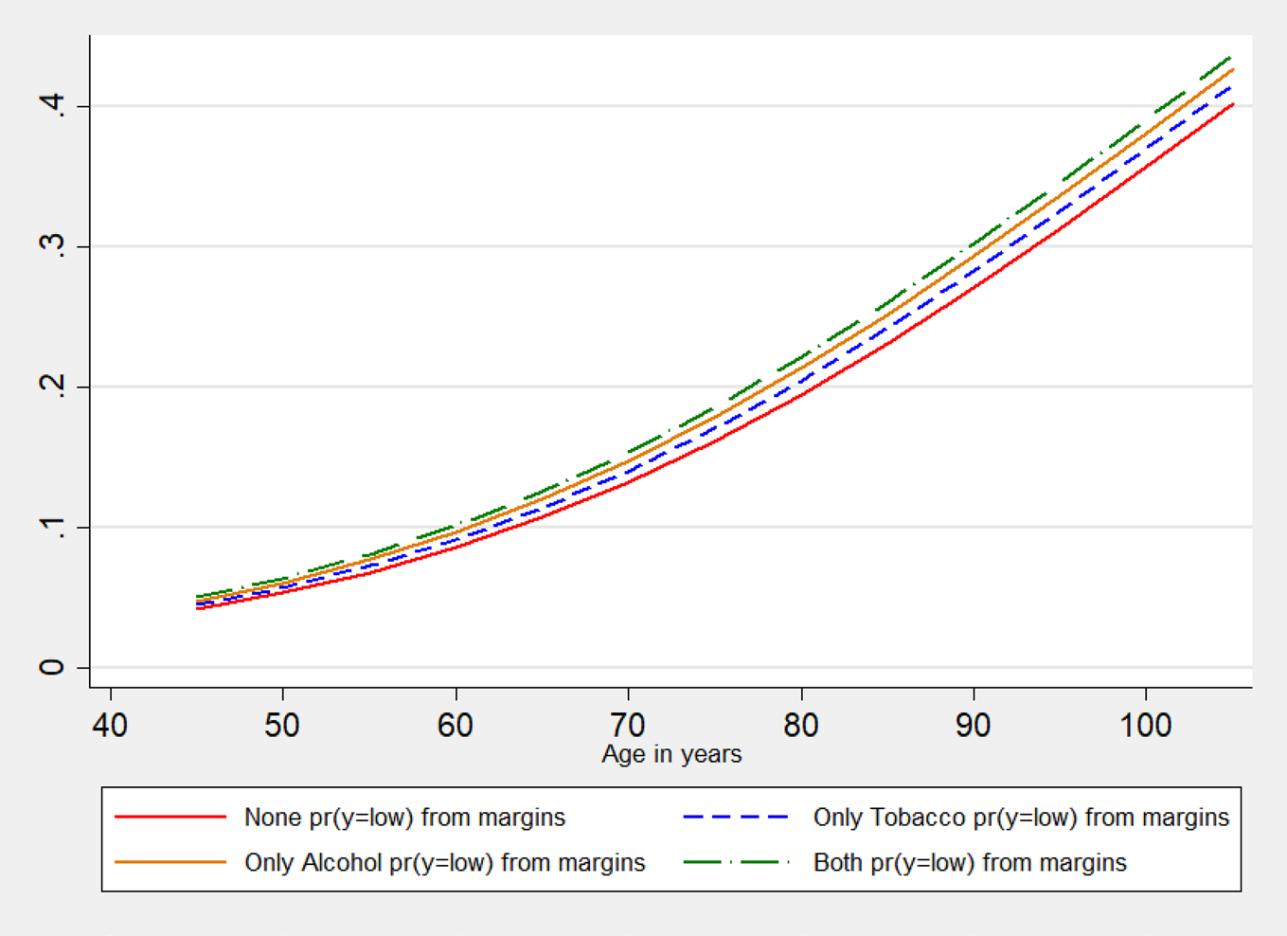
Average marginal effects of age on low cognition by substance use pattern among older adults, LASI, Wave 1, 2017–2018.

The average marginal effects of substance use patterns on low cognition are also computed and shown in Fig. 4. The graphical presentation of the marginal effects shows that individuals aged 45 and above who consume both alcohol and tobacco are exposed to a higher risk to experience low cognition followed by ‘only alcohol’ consumers and ‘only tobacco’ consumers. Although with increasing age older people are vulnerable to low cognitive capacity yet individuals who do not consume alcohol and tobacco carry the lowest chance to experience low cognition among others of the same age.

**Figure 5:**
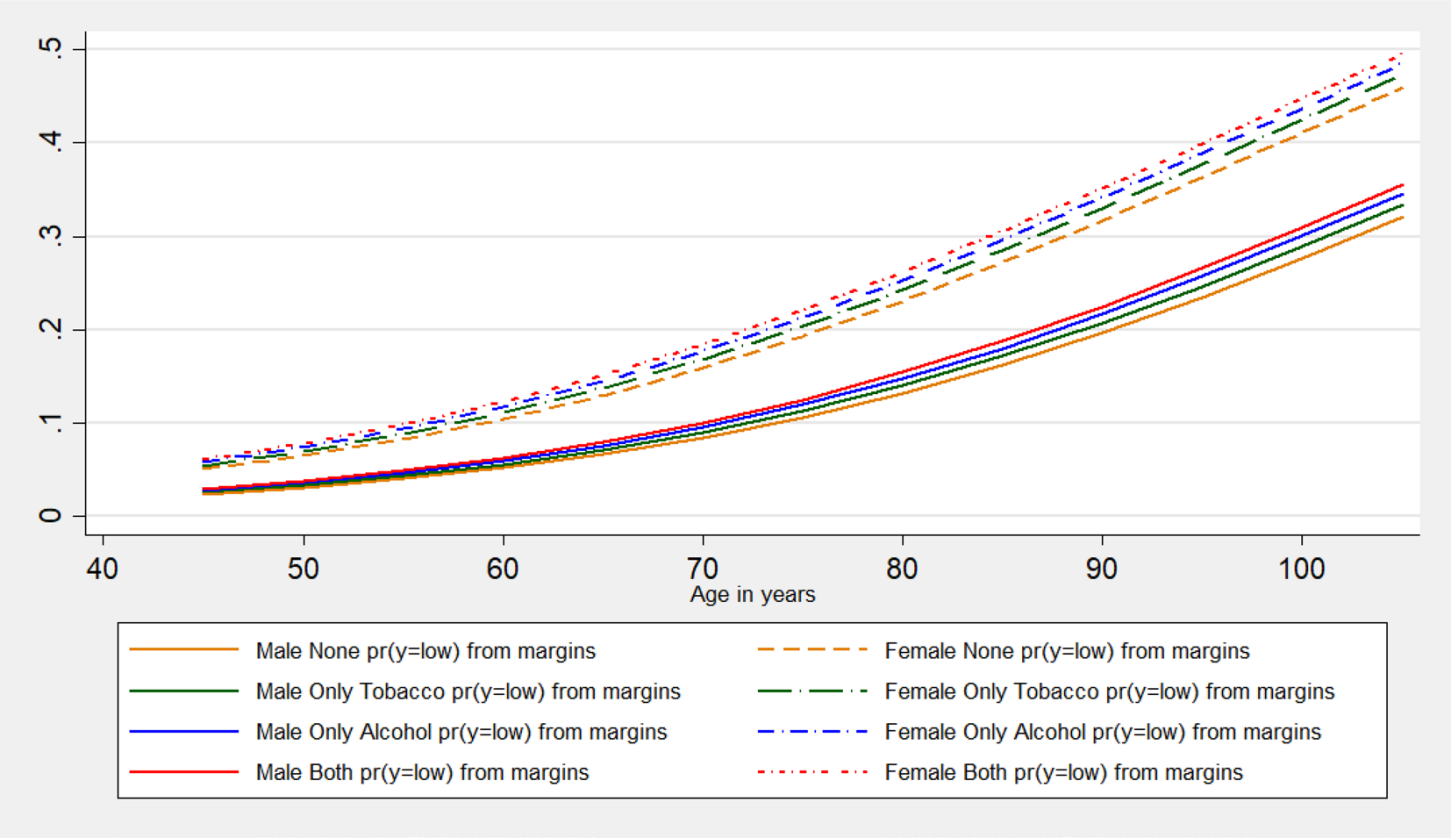
The gender pattern of average marginal effects of age on low cognition by substance use among older adults, LASI, Wave 1, 2017–2018.

From the male–female pattern of observed prevalence and the estimated odds ratio, it is clear that females of a particular age are always more likely and carry a higher chance to experience low cognition than the males of the same age. But it is also evident that substance use pattern shows a sex-specific variation in the marginal effects among individuals of different age. Overall, the risk of low cognition is highest among those who consume both tobacco and alcohol (Fig. 4). Exposure to alcohol as well as tobacco consumption makes both females as well as males carry the highest risk of experiencing low cognition than the rest of the categories of substance use pattern (Fig. 5). Among the other covariates, type of residence, marital status, educational attainment, caste, and economic well-being significantly predicts the cognition status of Indian older adults.

## Discussion

LASI is the first-ever survey conducted in India that offers comprehensive information on older adults aged 45 and above and their spouses. This is one nationally representative survey of a large and diverse sample of 72,250 older adults across India offering objective measures of height and weight to measure BMI, the information on health behaviour imparts the alcohol and tobacco consumption status of the individuals, the appropriate scale-based measures of several cognitive abilities functionally defines the cognition capacity, and the related socio-economic and demographic characteristics of the individuals provide the information on covariates. Thus, LASI eventually offers several advantages to investigate the relationship between health (BMI status) and health behaviour (Tobacco-alcohol consumption) with the cognitive function of older adults aged 45 and above. A partial-proportional odds model explains the relationship between the two key exposure variables and the cognitive function of older adults and demonstrates the associated risks of poor cognition in the study population. The study shows that BMI status and alcohol-tobacco consumption behaviour of the individuals strongly predict cognitive function among the older adults in the study population.

This study shows that cognitive capacity is strongly age-dependent and generally, older adults of higher age show poor cognitive capacity among them. From the analysis, it is evident that with increasing age, the probability to experience lower cognition shows a monotonic increase and the rate is also higher after age 60. Subject to the predominant age pattern of cognitive capacity, this study also clearly brings forth the evidence on BMI and tobacco-alcohol consumption status associated differential in cognition among older adults in India. It is observed that a significant portion of older adults (21%) are underweight and around 27% are overweight in India (Online Appendix S2). At the same time, almost two-fifth of the older adults consume tobacco (either smoke or smokeless) and around 15% of the older adults drink alcohol (Online Appendix S2).

In this study, it is found that the prevalence of low cognition is 18% among underweight compared to 5% among overweight and obese older adults aged 45 and above. The age pattern of cognition by BMI status demonstrates that the prevalence of low cognition is higher among underweight older adults aged 60 and above compared to the underweight older adults aged between 45 and 59 for both the sex. And in both age groups, underweight females show higher prevalence of low cognition. This hints that cognition is highly age dependent among Indian older adults, cognitive ageing is inevitable with growing age and female older adults are more vulnerable to low cognitive capacity with increasing age and undernutrition. The study findings indicate that female older adults are at higher risk of poor cognition compared the males and the findings are also in line with the previous study findings^45,46^. Similar to BMI status, health behaviour in terms of alcohol and tobacco consumption also shows a differential in cognitive capacity among Indian older adults. The multivariate estimation confirms that those individuals who consume both tobacco and alcohol are at higher risk of demonstrating lower cognition among them followed by ‘only alcohol’ consumers and ‘only tobacco consumers’.

A previous study evaluates cognitive impairment among older persons with respect to BMI status of the individuals and finds that BMI status is associated with mild cognitive impairment; whereby, gender and age play a moderating role^47^. Malnutrition is a potential risk factor for cognitive impairment among older persons and low BMI being an indicator of poor nutritional health can lead to cognitive impairment^48^. It has also been studied that high BMI during old age plays a protective role against the progression of dementia^49,50^. Another study reports that men who consumed 36 g/day of alcohol demonstrated a faster decline in all cognitive domains^51^. Though the pathway is complex, some previous studies also report that moderate alcohol consumption is associated with slower cognitive decline^52–55^. It is also evident that there is an underlying gender pattern in cognitive decline subject to alcohol consumption in the older population^51,56^. Another study, that examines the smoking-associated cognitive decline in early old age finds that middle-aged male smokers are at higher risk of experiencing faster cognitive decline than non-smokers^57^. On the other hand, for those who stopped smoking, cessation duration plays a role in cognitive decline^57^. The gender pattern of smoking is also attributable to smoking-associated differential in cognition among men and women; while smoking as a habit clusters differently with other risk factors among men and women to determine cognitive status among them^57,58^.

A previous study based on a panel dataset examined the associations between alcohol consumption patterns and dietary behaviours with cognitive impairment among Chinese older adult men and women and reported that former alcohol use was a risk factor for cognitive impairment among men only whereas light or heavy alcohol consumption was not associated with cognitive impairment^59^. Different other studies demonstrate mixed evidence on the same and has found that low to moderate alcohol consumption is a protective factor of cognitive function among older adults^52,60,61^ whereas, heavy alcohol consumption is associated with the increased risk of cognitive impairment^62,63^.

This study has several strengths. First, this study conducted a secondary analysis using the dataset from LASI survey which is country representative. Second, as this study is based upon a nationally representative large sample dataset, it has essentially helped to generalise the findings considering the Indian context of the older population. Lastly, as the statistical analyses are based upon a large number of samples, this study produces the estimates with enough statistical power while performing nutrition-specific (BMI status) and substance use-specific (health behaviour) estimations of the prevalence and risk of low cognitive function in the study population.

Nevertheless, this study has some limitations. First, due to the nature of the survey, this study could not infer the causal relationship between the study variables. Though nutritional status and health behaviour in terms of tobacco and alcohol consumption are independent risk factors of cognitive capacity among older adults with an underlying effect of age and sex, the results could still be subjected to residual confounding due to non-adjustment in the duration and intensity/pattern of substance use and change in BMI status over time. It is always recommended to adjust for the socioeconomic factors to get an unbiased estimate of the effect within a cross-sectional framework, but cross-sectional studies fail to overcome the cohort effects.

To note, LASI completed the first wave of the data collection only during 2017–2018 and this study produces the estimates based on one time-point of the dataset available from the survey and essentially the study is cross-sectional in nature. In this regard, a longitudinal survey collecting the panel information over time is advantageous and more effective to study chronic conditions like cognitive decline^59^ given nutritional status, alcohol and or tobacco consumption behaviour of a population with time. In this context, the exploration of this study topic will bring more analytical and practical evidence on the same once LASI completes collecting the information during future rounds of the survey.

## Conclusion

This study demonstrates that the older population in India is vulnerable to poor cognitive function and the risk of experiencing low cognition increases with increasing age among them. Though cognitive function is age dependent with an underlying gender difference, nutritional health and health (tobacco-alcohol consumption) behaviour also predicts cognitive capacity among older persons. The older population who are underweight carries a higher risk of low cognitive capacity, compared to those with normal and higher BMI (overweight and obese). Underweight female older adults are at substantially higher risk of experiencing low cognition compared to the underweight male older adults. As nutrition of older adults, does not get much attention, Government interventions and schemes should target the underweight older population, especially the women who are deprived, widowed or staying alone. Similar to nutritional health, health behaviour in terms of alcohol and tobacco consumption behaviour is also associated with the low cognitive capacity among Indian older adults and those who consume alcohol/tobacco or both are at higher risk of experiencing low cognitive capacity and the risk is very high in the higher ages. Again, the female older population is more vulnerable with a higher risk of experiencing low cognition subject to their alcohol-tobacco consumption behaviour, compared to the male population. As the study findings are indicative of the effects of tobacco and alcohol consumption on cognitive function, robust community-level awareness programs on the ill effects of alcohol-tobacco consumption can potentially help to deal with these behavioural risk factors of low cognition among the older population. Essentially, the intervention should be evidence-based and effective in substance use prevention. Along with the periodic campaigns, the intervention should also look after the supply–demand side of substance use across the locality.

## Supplementary Information

Supplementary Information.

## Data Availability

The data is available subject to a data request. The request can be put through the link https://www.iipsindia.ac.in/content/lasi-wave-i.
