# Author Correction: Nutritional status, alcohol-tobacco consumption behaviour and cognitive decline among older adults in India

**DOI:** 10.1038/s41598-023-27849-0

**Published:** 2023-01-16

**Authors:** Junaid Khan

**Affiliations:** 1 International Institute for Population Sciences, Deonar, Mumbai, 400088 India; 2 School of Public Health, Asian Institute of Public Health University, Bhubaneswar, 752101 India

Correction to: *Scientific Reports* 10.1038/s41598-022-25563-x, published online 06 December 2022

The original version of this Article contained an error in the Data availability section.

“The datasets analysed during the current study are available in the Demographic Health Survey (DHS) data repository, [https://dhsprogram.com/data/available-datasets.cfm].”

now reads:

“The data is available subject to a data request. The request can be put through the link https://www.iipsindia.ac.in/content/lasi-wave-i”.

The original Article has been corrected.

