# Supplementary material for: Nutritional status, alcohol-tobacco consumption behaviour and cognitive decline among older adults in India
Source: Sci Rep. 2022 Dec 6;12:21102. doi: 10.1038/s41598-022-25563-x (PMC9726887; doi:10.1038/s41598-022-25563-x)
Supplement: Supplementary file 1 — Supplementary Information. [file 41598_2022_25563_MOESM1_ESM.docx]

**Appendix 1: Socio-economic and demographic profile of the older adults aged 45 and above, India, 2017-18**

| **Background Characteristics** | **Distribution (%)** | **N** |
| --- | --- | --- |
| **Residence** |  |  |
| Rural | 65.1 | 34,301 |
| Urban | 34.9 | 18,364 |
| **Sex** |  |  |
| Male | 46.3 | 24,400 |
| Female | 53.7 | 28,265 |
| **Age** |  |  |
| 45-59 | 53.2 | 28,014 |
| 60+ | 46.8 | 24,651 |
| **Living arrangement** |  |  |
| Living alone | 3.5 | 1,828 |
| Living with Spouse and other | 15.3 | 8,066 |
| Living with Spouse and Children | 58.8 | 30,974 |
| Living with Children and other | 18.5 | 9,728 |
| Living with other | 3.9 | 2,069 |
| **Marital status** |  |  |
| Currently Married | 75.5 | 39,763 |
| Widowed | 21.4 | 11,269 |
| Other | 3.1 | 1,632 |
| **Caste** |  |  |
| Scheduled Caste | 17.4 | 8,812 |
| Scheduled Tribe | 18.6 | 9,444 |
| Other Backward Class | 38.5 | 19,505 |
| None | 25.6 | 12,967 |
| **Education** |  |  |
| Up-to Primary | 46.9 | 13,308 |
| Secondary | 35.0 | 9,919 |
| Higher and Above | 18.1 | 5,130 |
| **MPCE** |  |  |
| Poorest | 19.5 | 10,288 |
| Poor | 20.1 | 10,578 |
| Middle | 20.2 | 10,623 |
| Rich | 20.3 | 10,687 |
| Richest | 19.9 | 10,489 |
| **Region** |  |  |
| North | 24.1 | 12,683 |
| East | 18.4 | 9,694 |
| North-east | 13.8 | 7,255 |
| South | 23.9 | 12,562 |
| Central | 6.5 | 3,427 |
| West | 13.4 | 7,044 |
| **Total** | **100** | **52,665** |

**Appendix 2: Nutritional status, consumption pattern of alcohol, tobacco and cognitive profile of the study population aged 45 and above in India, 2017-18**

| **Key predictor** | **Total Population** | | **Men (%)** | | **Women (%)** | |
| --- | --- | --- | --- | --- | --- | --- |
| **variables** | **45+**  **(%)** | **N** | **45-59**  **%** | **60+**  **%** | **45-59**  **%** | **60+**  **%** |
| **BMI Status** | |  |  |  |  |  |
| Underweight | 21.21 | 9,598 | 16.23 | 28.16 | 15.73 | 25.28 |
| Normal | 51.18 | 27,516 | 57.47 | 53.66 | 46.68 | 48.43 |
| Overweight | 20.81 | 11,591 | 22.01 | 15.24 | 25.98 | 19.09 |
| Obese | 6.81 | 3,960 | 4.29 | 2.93 | 11.62 | 7.2 |
| **Alcohol consumption** | | |  |  |  |  |
| Never | 84.89 | 43,240 | 68.44 | 71.77 | 97.31 | 97.44 |
| Infrequent non-heavy | 9.12 | 5,423 | 18.11 | 18.35 | 1.45 | 1.37 |
| Frequent non-heavy | 3.02 | 1,962 | 6.73 | 5.01 | 0.66 | 0.59 |
| Heavy | 2.96 | 2,040 | 6.71 | 4.87 | 0.58 | 0.59 |
| **Tobacco consumption** | | |  |  |  |  |
| Never | 61.72 | 33,065 | 41.57 | 38.93 | 82.85 | 76.62 |
| Smoked | 14.2 | 7,889 | 25.93 | 28.39 | 2.29 | 4.24 |
| Smokeless | 20.96 | 9,889 | 26.53 | 25.92 | 14.59 | 18.65 |
| Both | 3.12 | 1,822 | 5.98 | 6.76 | 0.26 | 0.48 |
| **Alcohol and Tobacco consumption** | | |  |  |  |  |
| None | 58.62 | 30,646 | 35.33 | 34.32 | 81.71 | 75.48 |
| Only Tobacco | 25.98 | 12,421 | 32.45 | 36.98 | 15.51 | 21.9 |
| Only Alcohol | 3.1 | 2,419 | 6.23 | 4.62 | 1.14 | 1.14 |
| Both | 12.3 | 7,179 | 25.98 | 24.08 | 1.63 | 1.48 |
| **Outcome variable** | |  |  |  |  |  |
| **Cognition Score*** | |  |  |  |  |  |
| Low | 10.19 | 5,297 | 2.47 | 7.61 | 8.16 | 21.53 |
| Medium | 74.11 | 38,111 | 68.28 | 76.89 | 77.59 | 72.81 |
| High | 15.7 | 9,257 | 29.26 | 15.5 | 14.25 | 5.66 |
| **Total** | **100** | **52,665** | **100** | **100** | **100** | **100** |

*Details of the cognition score is described in the methods section

**Appendix 3: Ordered logistic estimation of cognitive capacity among older adults aged 45 and above, India, LASI Wave 1, 2017-18**

| **Background Chs.** | Cognition | | |
| --- | --- | --- | --- |
|  | AOR | p-value | 95% CI |
| **BMI Status** |  |  |  |
| Normal |  |  |  |
| Underweight | 0.68 | 0.000(<.01) | 0.64-0.72 |
| Overweight | 1.28 | 0.000(<.01) | 1.21-1.35 |
| Obese | 1.35 | 0.000(<.01) | 1.25-1.47 |
| **Substance use** |  |  |  |
| No tobacco, no alcohol |  |  |  |
| Tobacco only | 0.92 | 0.002 | 0.87-0.97 |
| Alcohol only | 0.85 | 0.001 | 0.77-0.94 |
| Both | 0.83 | 0.000(<.01) | 0.78-0.89 |
| **Age** |  |  |  |
| 45-59 |  |  |  |
| 60+ | 0.52 | 0.000(<.01) | 0.50-0.55 |
| **Residence** |  |  |  |
| Rural |  |  |  |
| Urban | 1.56 | 0.000(<.01) | 1.49-1.64 |
| **Sex** |  |  |  |
| Male |  |  |  |
| Female | 0.59 | 0.000(<.01) | 0.56-0.62 |
| **Living arrangement** |  |  |  |
| Living alone |  |  |  |
| Living with Spouse and other | 0.92 | 0.418 | 0.75-1.13 |
| Living with Spouse and Children | 1.04 | 0.708 | 0.85-1.27 |
| Living with Children and other | 1.12 | 0.068 | 0.99-1.26 |
| Living with other | 0.89 | 0.135 | 0.76-1.04 |
| **Marital status** |  |  |  |
| Currently Married |  |  |  |
| Widowed | 0.60 | 0.000(<.01) | 0.50-0.72 |
| Other | 0.96 | 0.693 | 0.77-1.19 |
| **Caste** |  |  |  |
| Scheduled Caste |  |  |  |
| Scheduled Tribe | 0.59 | 0.000(<.01) | 0.54-0.63 |
| Other Backward Class | 1.17 | 0.000(<.01) | 1.10-1.24 |
| None | 1.03 | 0.369 | 0.96-1.10 |
| **Education** |  |  |  |
| No education |  |  |  |
| Upto Primary | 3.88 | 0.000(<.01) | 3.63-4.15 |
| Secondary | 9.21 | 0.000(<.01) | 8.55-9.91 |
| Higher and Above | 17.79 | 0.000(<.01) | 16.29-19.43 |
| **MPCE** |  |  |  |
| Poorest |  |  |  |
| Poor | 1.09 | 0.010 | 1.02-1.17 |
| Middle | 1.24 | 0.000(<.01) | 1.16-1.33 |
| Rich | 1.33 | 0.000(<.01) | 1.24-1.42 |
| Richest | 1.52 | 0.000(<.01) | 1.42-1.63 |
| **Region** |  |  |  |
| North |  |  |  |
| East | 1.12 | 0.001 | 1.05-1.19 |
| North-east | 1.31 | 0.000(<.01) | 1.21-1.42 |
| South | 1.52 | 0.000(<.01) | 1.43-1.62 |
| Central | 1.25 | 0.000(<.01) | 1.14-1.37 |
| West | 0.79 | 0.000(<.01) | 0.73-0.85 |

**Appendix 4: Generalised ordered logistic estimation of cognition capacity among older adults aged 45 and above, India, LASI Wave 1, 2017-18**

| **Background chs.** | Low vs Medium & High | | | Low & Medium vs High | | |
| --- | --- | --- | --- | --- | --- | --- |
|  | AOR | p-value | 95% CI | AOR | p-value | 95% CI |
| **BMI Status** |  |  |  |  |  |  |
| Normal |  |  |  |  |  |  |
| Underweight | 0.70 | 0.000(<.01) | 0.65-0.75 | 0.67 | 0.000(<.01) | 0.61-0.74 |
| Overweight | 1.46 | 0.000(<.01) | 1.32-1.62 | 1.22 | 0.000(<.01) | 1.14-1.29 |
| Obese | 1.49 | 0.000(<.01) | 1.25-1.78 | 1.29 | 0.000(<.01) | 1.18-1.42 |
| **Substance use** |  |  |  |  |  |  |
| No tobacco, no alcohol |  |  |  |  |  |  |
| Tobacco only | 0.94 | 0.144 | 0.87-1.02 | 0.90 | 0.005 | 0.84-0.97 |
| Alcohol only | 0.82 | 0.015 | 0.70-0.96 | 0.91 | 0.133 | 0.81-1.03 |
| Both | 0.83 | 0.002 | 0.74-0.93 | 0.84 | 0.000(<.01) | 0.77-0.91 |
| **Age** |  |  |  |  |  |  |
| 45-59 |  |  |  |  |  |  |
| 60+ | 0.42 | 0.000(<.01) | 0.39-0.46 | 0.61 | 0.000(<.01) | 0.57-0.64 |
| **Residence** |  |  |  |  |  |  |
| Rural |  |  |  |  |  |  |
| Urban | 1.56 | 0.000(<.01) | 1.43-1.70 | 1.54 | 0.000(<.01) | 1.46-1.63 |
| **Sex** |  |  |  |  |  |  |
| Male |  |  |  |  |  |  |
| Female | 0.51 | 0.000(<.01) | 0.47-0.55 | 0.64 | 0.000(<.01) | 0.60-0.69 |
| **Living arrangement** |  |  |  |  |  |  |
| Living alone |  |  |  |  |  |  |
| Living with Spouse and other | 0.93 | 0.717 | 0.65-1.35 | 0.84 | 0.168 | 0.65-1.08 |
| Living with Spouse and Children | 1.02 | 0.897 | 0.72-1.47 | 0.99 | 0.919 | 0.77-1.27 |
| Living with Children and other | 1.07 | 0.371 | 0.92-1.23 | 1.08 | 0.391 | 0.90-1.30 |
| Living with other | 0.93 | 0.475 | 0.78-1.13 | 0.76 | 0.020 | 0.61-0.96 |
| **Marital status** |  |  |  |  |  |  |
| Currently Married |  |  |  |  |  |  |
| Widowed | 0.57 | 0.001 | 0.40-0.80 | 0.71 | 0.002 | 0.56-0.88 |
| Other | 0.63 | 0.017 | 0.43-0.92 | 1.21 | 0.137 | 0.94-1.56 |
| **Caste** |  |  |  |  |  |  |
| Scheduled Caste |  |  |  |  |  |  |
| Scheduled Tribe | 0.56 | 0.000(<.01) | 0.50-0.62 | 0.67 | 0.000(<.01) | 0.60-0.74 |
| Other Backward Class | 1.14 | 0.005 | 1.04-1.24 | 1.21 | 0.000(<.01) | 1.11-1.32 |
| None | 1.11 | 0.046 | 1.00-1.24 | 1.03 | 0.463 | 0.95-1.13 |
| **Education** |  |  |  |  |  |  |
| No education |  |  |  |  |  |  |
| Upto Primary | 3.77 | 0.000(<.01) | 3.42-4.16 | 3.72 | 0.000(<.01) | 3.41-4.06 |
| Secondary | 15.86 | 0.000(<.01) | 12.34-20.38 | 8.86 | 0.000(<.01) | 8.12-9.66 |
| Higher and above | 32.81 | 0.000(<.01) | 18.53-58.12 | 17.64 | 0.000(<.01) | 15.96-19.49 |
| **MPCE** |  |  |  |  |  |  |
| Poorest |  |  |  |  |  |  |
| Poor | 1.15 | 0.003 | 1.05-1.25 | 1.02 | 0.624 | 0.93-1.12 |
| Middle | 1.25 | 0.000(<.01) | 1.14-1.38 | 1.20 | 0.000(<.01) | 1.10-1.31 |
| Rich | 1.42 | 0.000(<.01) | 1.28-1.57 | 1.25 | 0.000(<.01) | 1.14-1.37 |
| Richest | 1.45 | 0.000(<.01) | 1.29-1.62 | 1.49 | 0.000(<.01) | 1.36-1.64 |
| **Region** |  |  |  |  |  |  |
| North |  |  |  |  |  |  |
| East | 1.01 | 0.874 | 0.91-1.11 | 1.21 | 0.000(<.01) | 1.11-1.32 |
| North-east | 1.01 | 0.927 | 0.89-1.13 | 1.55 | 0.000(<.01) | 1.40-1.72 |
| South | 1.27 | 0.000 | 1.14-1.40 | 1.69 | 0.000(<.01) | 1.56-1.83 |
| Central | 1.04 | 0.604 | 0.90-1.20 | 1.44 | 0.000(<.01) | 1.28-1.63 |
| West | 0.68 | 0.000(<.01) | 0.61-0.76 | 0.89 | 0.011 | 0.81-0.97 |
